# Short intense psychological stress induced by skydiving does not impair intestinal barrier function

**DOI:** 10.1371/journal.pone.0254280

**Published:** 2021-07-08

**Authors:** Maria Fernanda Roca Rubio, Ulrika Eriksson, Robert J. Brummer, Julia König

**Affiliations:** 1 Nutrition-Gut-Brain Interactions Research Centre, Faculty of Medicine and Health, School of Medical Sciences, Örebro University, Örebro, Sweden; 2 Man-Technology-Environment Research Centre, School of Science and Technology, Örebro University, Örebro, Sweden; McMaster University, CANADA

## Abstract

**Background and aim:**

Psychological stress has been shown to increase intestinal permeability and is associated with the development of gastrointestinal disorders. This study aimed to investigate skydiving as an alternative model to analyse the effect of acute psychological stress on intestinal barrier function.

**Materials and methods:**

Twenty healthy subjects participated in a tandem skydive followed by a negative control visit, of which 19 (9 females and 10 males, 25.9 ± 3.7 years) were included in the study. Intestinal permeability was assessed by a multi-sugar urinary recovery test. Sucrose recovery and lactulose/rhamnose ratio in 0-5h urine indicated gastroduodenal and small intestinal permeability, respectively, and sucralose/erythritol ratio in 5-24h urine indicated colonic permeability. Blood samples were taken to assess markers associated with barrier function. This study has been registered at ClinicalTrials.gov (NCT03644979) on August 23, 2018.

**Results:**

Skydiving resulted in a significant increase in salivary cortisol levels directly after skydiving compared to the control visit. Cortisol levels were still increased two hours after landing, while cortisol levels before skydiving were not significantly different from the baseline at the control visit. Skydiving did not induce a significant increase in gastroduodenal, small intestinal or colonic permeability. There was also no significant increase in plasma intestinal and liver fatty acid-binding proteins, suggesting no damage to the enterocytes.

**Discussion:**

These results show that the acute intense psychological stress induced by skydiving does not affect intestinal permeability in healthy subjects. Future models aiming to investigate the effect of stress on human intestinal barrier function should consider a more sustained exposure to the psychological stressor.

## Introduction

The intestinal barrier, the largest interface between the external environment and the host, plays a crucial role in gut health and is an important part of the gut-brain axis. It provides a barrier that prevents translocation of harmful compounds, while at the same time maintaining homeostasis with our gut microbial ecosystem [1]. Disruption of this barrier can result in an increased intestinal permeability, which may lead to translocation of pathogenic bacteria into the blood stream and local as well as systemic immune activation. Increased intestinal permeability has been associated with several diseases and disorders such as coeliac disease, inflammatory bowel diseases, irritable bowel syndrome and obesity [1, 2]. In addition, stress can lead to disturbance of the intestinal barrier, as shown in both animal models and in humans [3, 4]. Psychological stress can increase intestinal permeability via stimulation of the hypothalamic-pituitary-adrenal (HPA) axis and consequent intestinal mast cell activation [4]. The HPA axis is a major neuroendocrine system that controls acute and chronic stress reactions and acts as an essential component of the communication between the brain and the gut [5, 6]. Activation of the HPA axis starts with hypothalamic release of corticotrophin-releasing hormone (CRH), one of the key mediators of the stress response. CRH can reach the intestine, where it mediates its effects via local mast cell activation and degranulation [4, 7–9]. Upon activation, mast cells release a variety of pro-inflammatory mediators, such as proteases, all of which can negatively affect intestinal epithelial barrier function and can lead to disruption of the intestinal epithelial integrity [8, 10].

In humans, the effect of acute psychological stress on intestinal barrier function has been investigated using cold water immersion [11], combat training [12], public speech and anticipation of electroshocks [4]. Although cold water immersion is commonly used as a psychological stress model [13, 14], the relevance of acute cold exposure as a psychological stressor is debatable, and the model has been criticised for not being capable of inducing substantial HPA axis activation [15]. In the case of combat training, it is difficult to separate the effects of psychological and physical stress, as strenuous exercise is also known to increase intestinal permeability [16].

While the public speech task seems to be a more reliable model for psychological stress, it does not necessarily produce the same stress in everyone, especially under artificial test conditions. Hence, there is a need for alternative psychological stress models to challenge the intestinal barrier *in vivo*. These models could amongst others be used to assess the functionality of nutritional interventions aimed at strengthening a stress-induced barrier disruption or improving its resilience. Skydiving is known to trigger a physiological stress response, including activation of both the sympathetic autonomic nervous system, shown by an increase in heart and respiration rates [17], as well as the HPA axis [18]. Especially in novice skydivers, skydiving seems to induce a cortisol response across most participants. For example, Meyer et al. found that of the 29 first-time skydivers included in their study, all apart from one showed an increase in cortisol by more than 15% [18]. Even in experienced skydivers a consistent increase in cortisol in response to a skydive is reported [17–19]. Thus, in this study, we chose to assess skydiving as an alternative model to investigate acute psychological stress and its effect on the intestinal barrier function. Our results showed that the stress induced by skydiving did not seem sufficient to affect barrier function in our study group.

## Methods

### Ethical statement

The study was performed according to the Helsinki declaration and its revisions, and was approved by the Central Ethical Review Board of Uppsala, Sweden (registration number 2017/313/01). The study was performed at Örebro University in Örebro, Sweden, from July 2018 to October 2018. The trial has been registered at ClinicalTrials.gov (NCT03644979) on August 23, 2018. We did not consider this study as a traditional clinical trial or intervention study, as the aim was to elucidate the effect of the psychological stress induced by skydiving on intestinal permeability. In addition, this study did not include a patient group. This resulted in a delay in registration of this study. The authors confirm that there are no other ongoing or related trials for this intervention, and that potential future trials will be registered prospectively. Written informed consent was obtained from all study participants before start of the study.

### Participants

Healthy subjects were recruited via advertisements in social media and at Örebro University. Interested individuals willing to perform a tandem skydive (connected to a harness attached to an instructor) for the first or second time received an informational email about the study and were invited for an information meeting held at Örebro University. After providing written informed consent, subjects were screened for eligibility in the study. Inclusion criteria were an age of 18–50 years, being novice tandem skydivers (not more than one previous tandem skydive) and willingness to refrain from probiotic products or medication known to alter gastrointestinal function throughout the study. Reasons for exclusion were history of gastrointestinal surgery or disease, hypertension, body weight over 100 kg or BMI over 35 kg/m^2^, recent or current use of medications (except oral contraceptives), recent or current disease or infection, major food allergies, smoking and/or chewing tobacco, planned change to current diet or exercise habits, pregnancy or breastfeeding, alcohol or substance misuse, and any other medical condition that could affect the experimental results. A detailed list of exclusion criteria is provided in S1 Table. Participants were compensated with 1500 SEK, corresponding to approximately half the price of a tandem skydive.

### Test conditions

In this study, 20 healthy subjects attended two visits, first the skydiving visit and, at least one week later, the control visit, i.e. subjects served as their own controls. We kept to this order as we expected participants to be nervous already some time before the actual skydiving, and as we intended to keep low stress levels at the control visit. In addition, with this outline, we were able to avoid bias due to diurnal hormonal changes and could have the respective sample collection times during the control visit at the same times of the day as during the skydiving visit. The latter was strongly dependent on wind and weather conditions, and not all participants were able to jump at the same time of the day. The skydiving visit took place at the local skydiving club in Örebro, Sweden (Örebro Fallskärmsklubb), while the control visit took place at Örebro University, Örebro, Sweden. The freefall during the actual skydive lasted approximately 45 seconds. It took approximately 30 minutes from boarding the plane to the actual jump, and then about 15 minutes from the opening of the parachute until landing. Study participants were asked to avoid alcohol, drugs, artificial sugars, spicy foods, and exertive exercise two days prior to and during each visit until urine collection for the *in vivo* multi-sugar permeability test (see below) was completed. In addition, participants were instructed to avoid caffeine-containing foods or drinks and intake of sugars included in the multi-sugar permeability test on test days. The evening before skydiving, participants recorded their food and fluid intake, and then were asked to consume the same foods and fluids on the evening before the control visit. In order to avoid extensive fasting times, two hours before the multi-sugar test, participants consumed a standardised snack (Clif Bar Chocolate chip, 257 kcal per bar, Clif Bar & Company, USA). We considered two hours sufficient time for digestion of the snack bar without interference with the sugars contained in the test sugar solution. Participants were asked to fast after intake of the multi-sugar solution until the end of the 5h urine collection, but to drink at least 1.5 litres of water. During the skydiving visit, participants consumed the multi-sugar solution directly before boarding the plane (circa 30 minutes before the skydive), followed by total urine collection for 5 and 24 hours. After landing, blood samples were collected.

During the control visit, participants were asked to rest and were allowed to collect saliva and urine samples at home, but had to come to the study centre for blood sample collection. Saliva and blood samples were collected at similar time points as during the skydiving intervention. In addition, the multi-sugar solution was consumed at a similar time point as during the skydiving visit, followed by total urinary collection for 5 and 24 hours.

### Salivary cortisol collection and assessment

Saliva samples were collected using Salivette collection tubes (Sarstedt, Germany) at the following time points: Two hours, one hour and 30 minutes before the skydive, directly after landing, as well as one hour and two hours after the skydive, respectively, and at corresponding times at the control visit. For saliva collection, participants placed the provided cotton swab in their mouth and kept it there for one minute before returning it into the Salivette collection tube. Saliva samples were stored frozen at -20°C until analysis. After thawing, Salivettes were centrifuged at 1,000g for 5 minutes, which resulted in a clear supernatant of low viscosity. Salivary cortisol concentrations were measured using a commercially available chemiluminescence immunoassay with high sensitivity according to the manufacturer’s instructions (RE52611, IBL-Hamburg, Germany). The intra- and inter-assay coefficients for cortisol concentration analysis were below nine percent.

### In vivo intestinal permeability test

Intestinal permeability was assessed using a multi-sugar urinary recovery test of orally administered water-soluble, non-metabolizable sugars that differ in size. This is a sensitive, non-invasive technique able to detect small changes in small and large intestinal permeability. The sugar molecules larger in size, such as lactulose and sucralose only pass the intestinal barrier paracellularly, and no active uptake occurs [20]. The smaller-sized molecules, such as rhamnose and erythritol, pass the barrier by transcellular passage, and provide a control for gastric emptying and dilution, intestinal transit time, absorption area, systemic distribution and renal function. The urinary recovery ratios are then applied to assess the intestinal permeability of the respective intestinal segments where absorption of the sugars occurs [1, 21–23]. Directly before boarding the plane, circa 30 minutes before the skydive, and at the corresponding time at the control visit, the participants consumed 150 ml of tap water containing 1 g sucrose (Nordic sugar, Sweden), 1 g lactulose (Solactis, France), 1 g sucralose (Bulk Powders, Sweden), 1 g erythritol (Ingredi, Sweden) and 0.5 g rhamnose (BioGaia, Sweden). This was followed by collection of all urine in provided urine jars (Sarstedt, Sweden) stored in cooling bags for 24 hours, divided into two fractions. The first fraction contained all urine from 0-5h and the second fraction all urine from 5-24h, respectively. During collection of fraction 1, the participants fasted but were asked to drink at least 1.5 litres of tap water. After finishing the 24h collection, both urine fractions were returned to the staff. Subsequently, aliquots from each jar were collected and stored at -80°C until further analyses. Gastroduodenal permeability was assessed by the urinary sucrose recovery in fraction 1 (0-5h), small intestinal permeability was assessed by the lactulose/rhamnose (L/R) recovery ratio in fraction 1 (0-5h), colonic permeability was assessed by the sucralose/erythritol (S/E) recovery ratio in fraction 2 (5-24h) [21, 22].

### Sample preparation and determination of sugar concentration

1 ml of urine was centrifuged at 21,000 g for 25 min at a temperature of 4°C. The supernatant was collected and the pellet discarded. 5 μL of ^13^C labelled internal standard (Sigma Aldrich, MO, USA) was added to 50 μl of urine. These aliquots were diluted to a final volume of 1 mL with 80:20 acetonitrile:water. Then, samples were centrifuged at 8000 g for 15 minutes. Analysis was conducted on an Acquity UPLC coupled to a Quattro Premier XE UPLC–MS/MS system (Waters Corporation, Milford, USA) with an atmospheric electrospray interface operating in negative ion mode. Separation of analytes was performed on an Acquity BEH Amide column (1.7 μm, 2.1 x 100 mm) (Waters Corporation, Milford, USA) with a column temperature of 50°C, injection volume of 10 μl, and a flow rate of 0.17 mL/min. An isocratic method was used with a mobile phase of 0.1% NH_4_OH in acetonitrile and water (70:30). In order to control for carry over and to monitor instrument stability, external standards and blanks were injected for every tenth sample. Mass analysis was conducted by monitoring two product ions for each of the respective sugars in multiple reaction monitoring mode (341.1>88.7 and 341.1>178.9 for sucrose, 341.16>100.80 and 341.16>160.80 for lactulose, 163.20>59.00 and 163.20>103.00 for rhamnose, 395.04>359 and 395.04>323.00 for sucralose, and 121.76>88.7 and 121.76>70.8 for erythritol). Quantification was done using an isotope dilution method with labelled internal standards and a five-point calibration curve. Procedure blanks were analysed along with the samples and as no presence of the analytes in the blanks was found, the lowest points in the calibration curve were used to assess the limits of detection. Samples out of the range of the calibration curve were diluted and re-analysed. In addition, an inhouse control sample was included in each batch.

### Measurement of plasma intestinal fatty acid-binding protein (I-FABP)

Plasma I-FABP concentrations were measured using an ELISA (HK406, HycultBiotech, Uden, The Netherlands) following the manufacturer’s instructions. The detection range of this assay was specified to be 47 to 3,000 pg/ml.

### Measurement of plasma liver fatty acid-binding protein (L-FABP)

Plasma L-FABP concentrations were measured using an ELISA (HK404, HycultBiotech) following the manufacturer’s instructions. The detection range of this assay was specified to be 102 to 25,000 pg/ml.

### Measurement of plasma lipopolysaccharide binding protein (LPB)

Plasma LBP concentrations were measured using an ELISA (HK315, HycultBiotech) following the manufacturer’s instructions. The detection range of this assay was specified to be 4.4 to 50 ng/ml.

### Sample size calculations

Based on previous results by Vanuytsel *et al*. which detected a change in intestinal permeability after a public speech challenge compared to control of 0.029, with a mean SD of 0.031 [4], and our own data (unpublished, difference in L/R ratio between rest and after 1 hour of strenuous exercise on L/R ratio: 0.02, mean SD of 0.026), we estimated that with a power of 80% and a 95% confidence interval, n = 13 subjects needed to be included. Considering an expected dropout rate of 20%, n = 15 subjects would have to be included. However, as the actual impact of skydiving on the intestinal permeability was unknown, we included n = 20 study participants.

### Data analysis

The Shapiro-Wilk test was used to test normality of the data sets. The non-parametric paired Wilcoxon signed-rank test was used to analyse differences in intestinal permeability between the test conditions. Data is shown as median and interquartile range (IQR). The salivary cortisol data was log2-transformed resulting in a normally distributed data set. As repeated measures analysis of variance (ANOVA) cannot handle missing values, saliva cortisol data was analysed by fitting a mixed effect model as implemented in GraphPad Prism 8.0, with test condition (skydiving or control) and sampling time (t_-120_, t_-60_, t_-30_, t_+5_, t_+60_, t_+120_) both being fixed factors. This mixed model uses a compound symmetry covariance matrix and Geisser-Greenhouse correction and is fitted using Restricted Maximum Likelihood (REML). Alpha was set at 0.05, and the Bonferroni test was used to correct for multiple comparisons. P-values were adjusted accordingly. Concentrations of salivary cortisol are shown in their original form (before log2 transformation) as median and IQR. Correlations between variables were assessed using two-tailed Spearman’s rank-order correlation with a Bonferroni correction using IBM SPSS Statistics for windows, version 26 (IBM Corp., Armonk, N.Y., USA). All other statistical calculations were prepared with GraphPad Prism 9.1.0 (GraphPad Software Incorporated, La Jolla, CA, USA). All relevant data produced and analysed for this manuscript is provided in the S2 Table.

## Results

### Subject characteristics

Twenty healthy subjects (10 females and 10 males) were included in this study. Data of one female participant was excluded, as she started with intake of selective serotonin re-uptake inhibitors (SSRIs) during the study phase. Mean age of included study participants (9 females, 10 males) was 25.9 years (SD 3.7) and mean body mass index was 23.7 kg/m^2^ (SD 2.6). The study was performed at Örebro University in Örebro, Sweden, from July 2018 to October 2018.

### Effect of short intense psychological stress induced by skydiving on salivary cortisol

**Fig 1:**
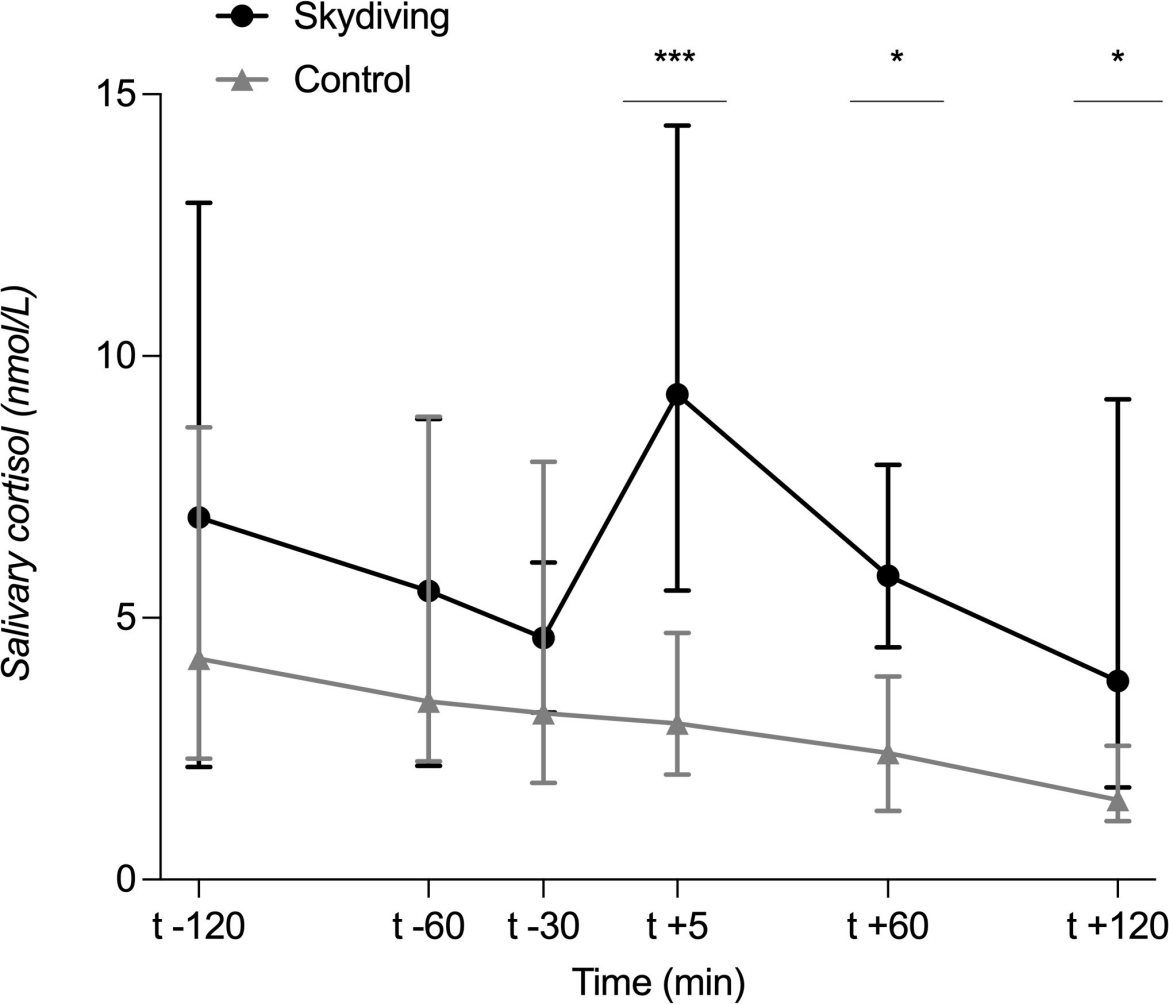
Salivary cortisol concentrations during the test conditions. Median and IQR are shown. p-values indicate significant differences between the two conditions. *p<0.05 (two hours after the skydive). *p<0.05 (one hour after the skydive). ***p<0.001 (directly after landing).

Cortisol responses to short intense psychological stress induced by skydiving are displayed in (Fig 1). The mixed-effects analysis showed a main effect of the test condition (skydiving or control) [F (1, 18) = 31.9; p<0.0001]. Time of sampling also had a statistically significant effect [F (3, 48) = 4.5; p<0.01]. We also observed a significant Condition x Time interaction [F (2, 37) = 3.4; p<0.05]. Follow-up tests (multiple comparisons) showed an increase in cortisol concentrations directly after the skydive (p<0.001) which remained significantly increased one hour (p<0.05) and two hours after (p<0.05) landing (Fig 1) in comparison to the corresponding times at the control visit. At baseline, salivary cortisol concentrations did not differ significantly between the control and skydiving conditions [Fig 1, control: 2h before (t_-120_): median of 4.2 nmol/l interquartile range (IQR) of 2.3–8.6 nmol/l; skydiving: 6.9 nmol/l, 2.1–12.9 nmol/l; adjusted p-value >0.999]. Salivary cortisol concentrations remained without significant changes one hour before (t_-60_) (control: 3.4 nmol/l, 2.3–8.8 nmol/l; skydiving: 5.2 nmol/l, 2.1–8.3 nmol/l; p>0.999) and 30 minutes before skydiving (t_-30_) (control: 3.2 nmol/l, 1.8–7.9 nmol/l; skydiving: 4.5 nmol/l, 2.9–6.7 nmol/l; p = 0.17). Directly after landing (t_+5_), salivary cortisol concentrations were significantly increased in comparison to the control condition (control: 2.9 nmol/l, 2.0–4.7 nmol/l; skydiving: 9.2 nmol/l, 4.7–14.3 nmol/l; p<0.001). Salivary cortisol levels after skydiving were still significantly increased one hour (t_+60_) (control: 2.4 nmol/l, 1.3–3.9 nmol/L; skydiving: 5.8 nmol/l, 4.4–7.9 nmol/l; p<0.05) and two hours after landing (t_+120_) (control: 1.7 nmol/l, 1.1–2.7 nmol/l; skydiving: 3.8 nmol/l, 1.8–9.2 nmol/l; p<0.05), respectively, compared to the control condition.

### Effect of short intense psychological stress induced by skydiving on intestinal permeability

**Fig 2:**
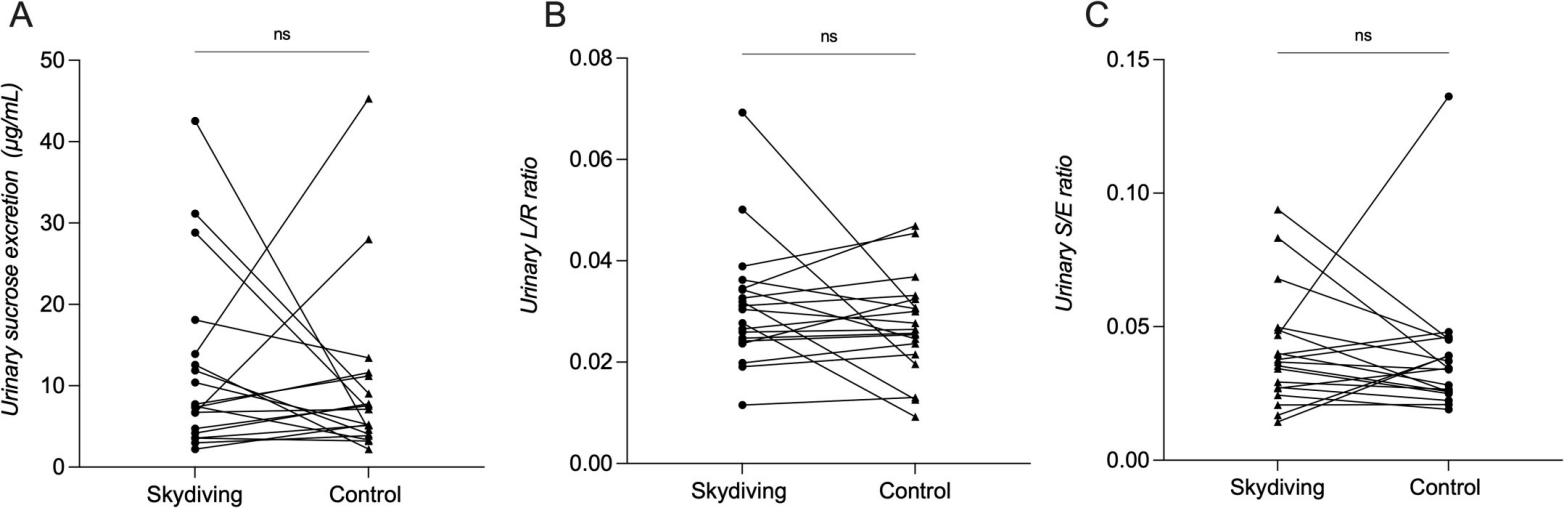
Intestinal permeability at the different test conditions. (A) Gastroduodenal permeability measured by urinary sucrose excretion (0-5h). (B) Small intestinal permeability measured by lactulose/rhamnose (L/R) ratio (0-5h). (C) Colonic permeability measured by urinary sucralose/erythritol (S/E) ratio (5h-24h). There was no significant difference among the test conditions.

Short intense psychological stress induced by skydiving was not associated with increased gastroduodenal permeability, assessed by urinary sucrose recovery (0-5h) (control visit: median of 7.1 μg/ml, IQR of 4.1–11.2 μg/ml; after skydiving: 7.5 μg/ml, 4.2–14.0 μg/ml, p = 0.49) (Fig 2A). Also, small intestinal permeability was not affected by skydiving, measured as the urinary lactulose/rhamnose (L/R) recovery ratio (0-5h) (control visit: 0.026, 0.022–0.032; after skydiving: 0.030, 0.024–0.034, p = 0.77) (Fig 2B). Furthermore, there was no significant effect of skydiving on colonic permeability, measured as sucralose/erythritol (S/E) recovery ratio (5-24h) (control visit: 0.034, 0.026–0.045; after skydiving: 0.037, 0.027–0.049, p = 0.31) (Fig 2C).

### Effect of short intense psychological stress induced by skydiving on other biomarkers of intestinal barrier function

**Fig 3:**
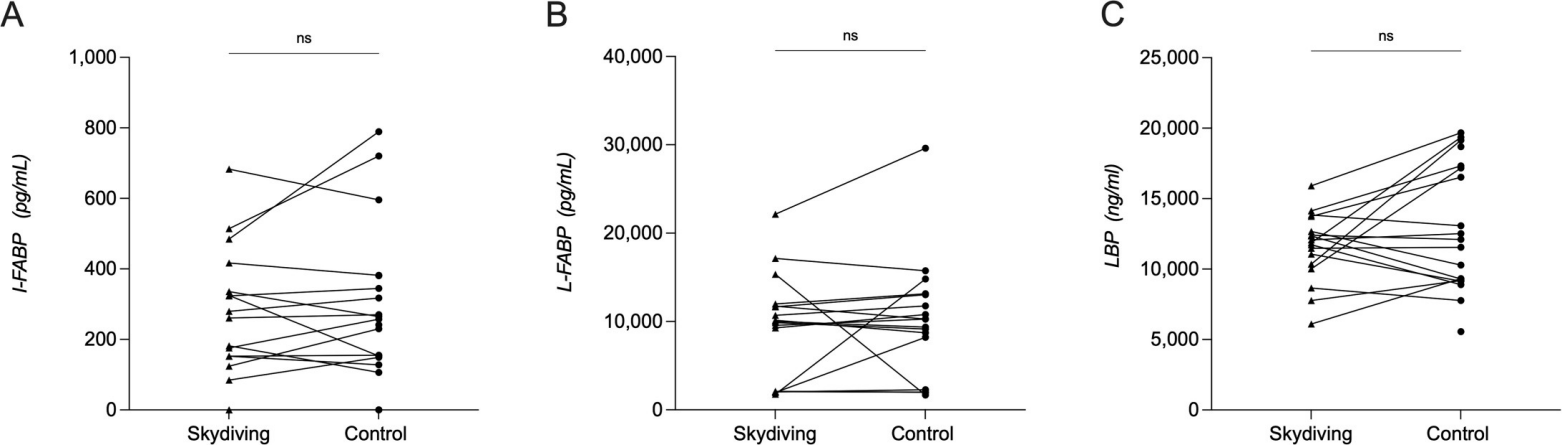
Concentrations of other biomarkers of intestinal barrier function at the different test conditions. (A) Plasma concentrations of intestinal fatty acid-binding protein (I-FABP). (B) Plasma concentrations of liver fatty acid-binding protein (L-FABP). There was no significant difference among the test conditions. (C). (LBP). There was no significant difference among the test conditions.

There was no significant difference in plasma intestinal fatty acid-binding protein (I-FABP) concentrations after the skydiving visit (median of 261 pg/ml, IQR of 138–376 pg/ml) compared to the control condition (257 pg/ml, 138–363 pg/ml, p = 0.59, Fig 3A). There was also no significant difference in plasma liver fatty acid-binding protein (L-FABP) concentrations after skydiving (10,004 pg/ml, 2,076–11,861 pg/ml) compared to the control condition (9,361 pg/ml, 2,253–13,033 pg/ml, p = 0.54) (Fig 3B). There was no significant difference in plasma LBP concentrations after skydiving (control visit: median of 12,104 ng/ml, IQR of 9,189–17,324 ng/ml; after skydiving: 11,871 ng/ml, 10,185–13,209 ng/ml, p = 0.17) (Fig 3C).

### Correlations between salivary cortisol levels during the skydiving visit and intestinal permeability assessed by the multi-sugar test

No significant correlations between small intestinal permeability (L/R recovery ratios) and the cortisol levels at any of the time points were found (L/R vs. cortisol, 2h before: r = -0.525, p = 0.02; 1h before: r = -0.298, p = 0.20; 30 min before: r = 0.105, p = 0.65; directly after landing: r = -0.134, p = 0.57; 1h after landing: r = -0.308, p = 0.18; 2h after landing: r = -0.023, p = 0.92. Differences were considered significant if p<0.007 (Bonferroni-corrected data).

Also large intestinal permeability (S/E ratio) did not significantly correlate with salivary cortisol concentrations (S/E vs. cortisol, 2h before: r = -0.451, p = 0.05; 1h before: r = -0.168, p = 0.47; 30 min before: r = 0.586, p = 0.007; directly after landing: r = 0.349, p = 0.13; 1h after: r = -0.065, p = 0.78; 2h after: r = 0.117, p = 0.62).

### Correlations between plasma markers at the skydiving condition and intestinal permeability assessed by the multi-sugar test

L/R recovery ratios did not significantly correlate with neither I-FABP nor L-FABP (r = -0.109, p = 0.68 and r = 0.024, p = 0.92, respectively), nor LBP (r = 0.329, p = 0.18). Also, S/E recovery ratios did not significantly correlate with neither I-FABP nor L-FABP (r = -0.168, p = 0.53 and r = -0.088, p = 0.72, respectively), nor LBP (r = 0.333, p = 0.17).

## Discussion

In this study we assessed skydiving as a model to investigate the effect of acute psychological stress on intestinal permeability in healthy subjects. Skydiving, a potentially life-threatening activity, has been demonstrated to be an ideal non-artificial psychological stressor for experimental studies [17–19, 24–26], especially as the actual risk of death is low (0.57 deaths per 100,000 jumps) with no deaths among tandem skydives according to a recent study in France [27]. The intense emotional stress experienced before, during and after a skydive suggested it to be a suitable model to study the effect of psychological stress on the human intestinal barrier. As a potentially life-threatening activity, skydiving should induce a more intense challenge than what could be expected from an artificial laboratory setting, especially as participants are at an actual risk of severe injury or death [26]. However, in the current study, even though there was a significant increase in salivary cortisol concentrations after skydiving, no effect on intestinal barrier function was found.

In this study, we used the multi-sugar urine recovery test of orally administered water-soluble, non-metabolizable sugars by van Wijck *et al*. to assess intestinal permeability. This is a commonly used non-invasive method to detect small changes in small and large intestinal permeability [20, 28]. Additionally, plasma concentrations of intestinal and liver fatty acid binding proteins (I-FABP and L-FABP) were assessed as surrogate markers for intestinal epithelial cell damage [28, 29]. In the intestine, I-FABP and L-FABP are particularly highly expressed in cells present on the tips of the villi [30, 31]. The villi are the initial site of epithelial damage, causing the release of FABP proteins into the systemic circulation upon injury [1, 2]. Furthermore, translocation of bacterial products from the intestine into the systemic circulation was measured via the concentrations of lipopolysaccharide-binding protein (LBP) in plasma. LBP is an acute phase protein which binds to lipopolysaccharides (LPS) on the outer membrane of bacteria, leading to a pro-inflammatory host response when LPS passes the epithelial barrier [28, 32]. A recent study attributed acceptable reliability to all of these markers of gut barrier integrity [33]. The stress induced by skydiving did not affect any of these markers in our study, suggesting that skydiving does not have a harmful effect on the intestinal barrier.

Salivary cortisol is an established non-invasive biomarker for stress that correlates closely with the free cortisol fraction in blood [34–36]. Our protocol induced a significant increase in cortisol levels directly after landing (median of 9.1 nmol/l) in comparison to the respective time at the control visit (5.8 nmol/l). This increase was still significant two hours after skydiving and indicates that skydiving was a suitable model to induce acute psychological stress. Increases of salivary cortisol by 1.5 nmol/l (or 15.5%) have been evaluated as valid stress-induced responses [37]. Similar to what previous studies have shown [18, 24, 26], salivary cortisol levels from 2 hours until 30 minutes before skydiving (directly before boarding the plane) were similar to those at the control condition. These low cortisol levels before the skydive and a rather short-term increase of cortisol afterwards indicate that the stress encountered by the participants was not sufficient to compromise the intestinal epithelial barrier.

In a study by Vanuytsel *et al*., the intestinal barrier of healthy subjects was tested under two different psychological stress conditions (public speech and anticipation of electroshocks) compared to a control condition. The public speech task consisted of a bachelor’s or master’s thesis defence, where participants presented their work, followed by an oral examination (duration of circa 45 minutes). The shock condition consisted of a protocol involving anticipation of painful electroshocks in a darkened room with a 30 minutes duration. In this study, an increase in salivary cortisol levels was reported already one hour before the public speech task in comparison to the control and the shock condition. During the shock condition, salivary cortisol levels remained similar to the control condition. Small intestinal permeability was assessed by lactulose/mannitol recovery ratio, showing a significant increase in small intestinal permeability after the speech task but not the shock condition [4]. This is another indication of that skydiving in our study, even though there was an activation of the HPA axis, did not provoke a stress response strong enough to affect the intestinal barrier.

In our study, participants served as their own controls. First, they attended the skydiving visit, and subsequently, after a wash-out period of at least one week, they attended the control visit. This was done as we had expected participants to be nervous already some time before the actual skydiving, which could have led to increased values already at the control visit. On the other hand, having the control visit after the skydive could have led to a carry-over effect. Hence, we applied a wash-out period of one week, as by this time cortisol levels should be back to normal [36, 38] and barrier function should be re-established [2, 39, 40]. Indeed, at the control visit, salivary cortisol levels as well as intestinal permeability at the control visit were in the normal range [20, 41]. This was also the case for I-FABP, L-FABP, and LBP [30, 32, 42]. In addition, as cortisol levels are known to, apart from an increase after awakening, gradually decrease during the course of the day [43, 44], we collected all samples at the control visits at the same time of the day as during the skydiving visit. This would not have been possible in the same extent if we had scheduled the control visit prior to the skydiving.

A major limitation of this study was that we did not assess the participants’ subjective emotions. Especially novice skydivers were reported to have increased levels of state anxiety already before the jump [18, 19, 25]. In addition, Meyer et al. found that subjective pre-jump anxiety predicted cortisol reactivity in novice skydivers and that higher post-jump happiness predicted faster cortisol recovery [18]. Nevertheless, a lack of concordance has been reported between subjective ratings and cortisol levels in experienced skydivers [18, 19]. In addition, in the public speech task by Vanuytsel et al. the increase in intestinal permeability was only present in those with a significant elevation of cortisol [4].

Further limitations were that did we not include a control group that did not skydive at all. Unfortunately, due to technical difficulties related to the altitude of the skydive, we did not manage to collect the heart rates of participants, which could have been a secondary marker to assess psychological stress. In addition, we could have missed the peak in cortisol levels as we only collected samples directly after landing and 60 minutes after landing. Furthermore, the selection of participants may have affected the results of our study, as skydiving possibly attracts a population of risk-seeking volunteers that might be more resilient to psychological stress. In addition, the tandem instructors were very skilled in making the participants feel safe and confident, which may have influenced the stress levels prior the skydive. Hence, it would be interesting to repeat this study with skydivers performing their skydive without instructor for the first time. Furthermore, it would be of interest to investigate how the intestinal permeability in certain patient groups, for example with stress-related gastrointestinal disorders such as IBS, would be affected by the acute stress induced by skydiving, ideally in comparison to a prospectively recruited healthy group to account for potential differences in subjective experience.

In conclusion, this study showed that short intense psychological stress did not increase intestinal permeability or damage the intestinal barrier in healthy participants. Future experimental models aiming to investigate the effect of psychological stress on human intestinal barrier function should possibly consider a more sustained exposure to the psychological stressor.

## Supporting information

S1 Table Exclusion criteria. (PDF)

S2 Table Data file. (PDF)
