# Supplementary material for: Short intense psychological stress induced by skydiving does not impair intestinal barrier function
Source: PLoS One. 2021 Jul 8;16(7):e0254280. doi: 10.1371/journal.pone.0254280 (PMC8266057; doi:10.1371/journal.pone.0254280)
Supplement: S1 Table — (PDF) [file pone.0254280.s001.pdf]

S1 Table: Exclusion criteria

1. Abdominal surgery which might influence gastrointestinal function, except appendectomy and cholecystectomy.
2. Current diagnosis of hypertension.
3. Current diagnosis of psychiatric disease.
4. Over 100kg or with a BMI over 35.
5. Systemic use of steroids in the last 6 weeks.
6. Use of antibiotics or antimicrobial medication in the last month.
7. Daily usage of NSAID in the last 2 months or incidental use in the last 2 weeks prior to screening.
8. Usage of medications that could affect the barrier function, except oral contraceptives, during the 14 days prior to screening.
9. Diagnosed inflammatory gastrointestinal disease.
10. Regular use of probiotics in the last 6 weeks.
11. Smoking and/or chewable tobacco.
12. Planned changes to current diet or exercise regime.
13. Use of laxatives, anti-diarrhetics, anti-cholinergics within last 4 weeks prior to screening.
14. Use of immunosuppressant drugs within last 4 weeks prior to screening.
15. Women: Pregnancy, lactation.
16. Abuse of alcohol or drugs.
17. Any disease/condition which in the investigator's opinion could interfere with the intestinal barrier function.
18. Any clinically significant disease/condition which in the investigator's opinion could interfere with the results of the trial.
