# Supplementary material for: Short intense psychological stress induced by skydiving does not impair intestinal barrier function
Source: PLoS One. 2021 Jul 8;16(7):e0254280. doi: 10.1371/journal.pone.0254280 (PMC8266057; doi:10.1371/journal.pone.0254280)
Supplement: S2 Table — (PDF) [file pone.0254280.s002.pdf]

S2 Table: Data file

Participant	Sex	Age (years)	Weight (kg)	Height (m)	BMI (kg/m2)
**1**	female	24	54	1,71	20,18
**2**	male	27	71	1,8	21,91
**3**	female	22	65	1,64	24,17
**4**	female	23	55	1,56	22,60
**5**	male	25	70	1,85	20,45
**6**	female	30	65	1,63	24,46
**7**	male	31	93	1,85	27,17
**8**	male	23	82	1,85	23,96
**9**	male	32	66	1,73	22,05
**10**	female	27	69	1,68	24,45
**11**	female	23	70	1,67	25,10
**12**	male	24	75	1,78	23,67
**13**	male	34	73	1,76	23,57
**14**	male	21	76	1,7	26,30
**15**	male	27	79	1,85	23,08
**16**	male	26	74	1,86	21,39
**17**	female	28	70	1,7	24,22
**18**	female	24	98	1,78	30,93
**19**	female	22	63	1,75	20,57

Supplemental Table 2: Data file

Participant	L/R ratio Control	L/R ratio Skidiving	S/E ratio Control	S/E ratio Skidiving	Sucrose (μg/ml) Control
**1**	0,0265	0,0259	0,0224	0,0273	13,4280
**2**	0,0237	0,0198	0,0265	0,0293	45,2900
**3**	0,0308	0,0693	0,0454	0,0939	2,2160
**4**	0,0092	0,0277	0,0391	0,0169	5,2140
**5**	0,0325	0,0237	0,0462	0,0377	11,6120
**6**	0,0332	0,0311	0,0257	0,0354	28,0260
**7**	0,0306	0,0362	0,0480	0,0395	4,5920
**8**	0,0257	0,0247	0,0190	0,0243	7,1140
**9**	0,0246	0,0343	0,0451	0,0679	5,1260
**10**	0,0277	0,0304	0,0281	0,0400	4,0820
**11**	0,0369	0,0327	0,0257	0,0488	3,3360
**12**	0,0454	0,0389	0,0344	0,0833	11,2200
**13**	0,0131	0,0115	0,0249	0,0343	9,0380
**14**	0,0196	0,0501	0,1362	0,0469	7,1200
**15**	0,0255	0,0241	0,0392	0,0144	5,2400
**16**	0,0469	0,0345	0,0209	0,0207	7,7800
**17**	0,0215	0,0191	0,0344	0,0269	7,5640
**18**	0,0300	0,0266	0,0340	0,0368	3,2060
**19**	0,0126	0,0319	0,0371	0,0498	3,8820

Supplemental Table 2: Data file

Participant	Sucrose (μg/ml) Skidiving	I-FABP (pg/ml) Control	I-FABP (pg/ml) Skidiving	L-FABP (pg/ml) Control	L-FABP (pg/ml) Skidiving
**1**	18,1100	241,3680	Missing	2252,666667	Missing
**2**	13,9120	317,3727	279,7420	1998,806667	2101,786667
**3**	12,5560	380,7387	Missing	1918,38	Missing
**4**	10,4080	344,6320	323,5607	2019,493333	2051,173333
**5**	7,3560	128,2270	152,2913	2288,18	2031,513333
**6**	6,6780	264,9367	335,7467	1673,133333	15343,80667
**7**	42,5300	230,5127	124,1810	14809,54667	1756,326667
**8**	6,7340	155,3500	152,4030	10272,96667	9558,54
**9**	3,5700	152,7950	324,7393	10277,32667	11739,69333
**10**	11,8740	Under detection	Under detection	9361,086667	10004,22667
**11**	7,4920	106,4240	181,9153	8724,1	10139,5
**12**	7,7380	149,4180	84,6860	13143,68	11981,91333
**13**	31,1440	257,7560	175,7700	11760,99333	10690,94
**14**	28,8140	Under detection	Under detection	8216,726667	1964,42
**15**	2,2200	270,2900	261,0740	9147,453333	9881,92
**16**	4,1920	382,0007	417,2093	15749,36	17150,78
**17**	4,7320	789,1327	484,8600	29604,24667	22133,52
**18**	3,5700	595,6007	683,0567	10792,74667	9275,2
**19**	2,9820	720,3707	514,2327	13032,89333	11658,92

Supplemental Table 2: Data file

Participant	LBP (ng/ml) Control	LBP (ng/ml) Skidiving	Cortisol (nmol/ml) Control - 120min	Cortisol (nmol/ml) Control - 60 min	Cortisol (nmol/ml) Control -30min
**1**	5556	Missing	38,1800	4,3800	3,33
**2**	9332	6109	7,4000	2,2600	1,52
**3**	18688,66667	Missing	1,4600	22,3500	11,41
**4**	11542,33333	11482,33333	24,5600	9,5900	3,86
**5**	10286,66667	12675,66667	5,3600	1,5100	10,59
**6**	17323,66667	14133,33333	2,4400	8,8400	7,98
**7**	13078,66667	13836,66667	2,7100	2,9800	2,32
**8**	12516	12079,66667	1,3000	1,9900	1,15
**9**	7773,666667	8659,666667	4,2200	2,5600	2,34
**10**	12103,66667	12406,33333	3,7200	1,5900	1,87
**11**	17173,33333	10022,66667	4,7500	10,2100	8,76
**12**	16514,66667	13742	3,5800	2,8300	3,18
**13**	8902	11730,66667	10,1900	9,9800	8,66
**14**	9292,333333	12358,66667	2,2200	2,4000	1,85
**15**	9189	7765	1,4600	1,1400	1,54
**16**	9152,5	11067,33333	8,6400	3,4000	1,8
**17**	19358,66667	11870,5	12,7700	7,1500	3,98
**18**	19150,33333	10347,66667	2,3100	4,0500	2,18
**19**	19672	15914,66667	7,7800	6,1200	4,14

Supplemental Table 2: Data file

Participant	Cortisol (nmol/ml) Control 5min	Cortisol (nmol/ml) Control 60min	Cortisol (nmol/ml) Control 120min	Cortisol (nmol/ml) Skydiving -120min	Cortisol (nmol/ml) Skydiving -60 min
**1**	3,69	2,42	1,52	Missing	4,34
**2**	2,91	1,26	1,32	31,22	7,26
**3**	9,94	4,65	4,05	2,15	2,91
**4**	2,81	1,59	1,51	20,25	5,79
**5**	15,76	8,33	4,1	52,44	32,68
**6**	7,96	4,46	Missing	12,11	22,07
**7**	3,42	2,98	1,01	3,75	2,15
**8**	1,08	0,21	0,2	2,24	1,93
**9**	3,04	1,16	2,48	1,22	2,18
**10**	2,67	1,36	1,05	1,44	2,03
**11**	4,71	2,47	1,3	12,4	17,22
**12**	1,86	8,47	2,95	1,87	2,37
**13**	6,87	Missing	Missing	14,72	8,27
**14**	2,34	2,52	1,85	4,28	5,83
**15**	1,61	1,5	1,18	6,92	3,02
**16**	2,01	1,54	1,98	6,86	6,02
**17**	3,51	2,85	1,97	7,51	10,37
**18**	1,64	1,13	0,69	1,96	1,82
**19**	2,98	3,3	2,64	7,53	5,24

Supplemental Table 2: Data file

Participant	Cortisol (nmol/ml) Skydiving -30min	Cortisol (nmol/ml) Skydiving 5min	Cortisol (nmol/ml) Skydiving 60min	Cortisol (nmol/ml) Skydiving 120min
**1**	Missing	1,66	4,79	12,93
**2**	4,44	14,69	4,84	1,97
**3**	8,73	17,97	4,32	9,59
**4**	3,62	6,33	Missing	Missing
**5**	19,62	14,11	Missing	Missing
**6**	10,11	9,27	3,41	2,81
**7**	3,23	12,21	6,06	14,03
**8**	1,47	4,58	0,33	0,71
**9**	6,06	11,38	Missing	Missing
**10**	4,88	15,26	36,6	28,98
**11**	4,79	9,05	6,39	2,32
**12**	14,02	2,99	6,25	3,89
**13**	4,62	23,13	14,75	3,7
**14**	3,19	4,72	2,31	1,15
**15**	2,14	Missing	20,2	7,93
**16**	2,4	3,38	5,55	1,56
**17**	4,93	9,75	8,39	5,13
**18**	2,22	8,55	5,42	1,69
**19**	3,83	7,38	6,51	3,96
